# ﻿Glacial history of *Saxifragawahlenbergii* (Saxifragaceae) in the context of refugial areas in the Western Carpathians

**DOI:** 10.3897/phytokeys.246.118796

**Published:** 2024-09-20

**Authors:** Elżbieta Cieślak, Michał Ronikier, Magdalena Szczepaniak

**Affiliations:** 1 W. Szafer Institute of Botany, Polish Academy of Sciences, Lubicz 46, PL-31-512 Kraków, Poland W. Szafer Institute of Botany, Polish Academy of Sciences Kraków Poland

**Keywords:** AFLP, haplotype, high mountain plant, narrow endemic, phylogeography, refugial areas, Tatra Mts

## Abstract

Despite the wealth of data available for mountain phylogeography, local-scale studies focused on narrow endemic species remain rare. Yet, knowledge of the genetic structure of such species biogeographically linked to a restricted area is of particular importance to understand the history of the local flora and its diversity patterns. Here, we aim to contribute to the phylogeographical overview of the Western Carpathians with a genetic study of *Saxifragawahlenbergii*, one of the most characteristic endemic species of this region. We sampled populations from all discrete parts of the species’ distribution range to apply sequencing of selected non-coding cpDNA and nuclear ribosomal DNA (ITS) regions, as well as Amplified Fragment Length Polymorphism (AFLP) fingerprinting. First, while ITS sequences showed weak diversification, the genetic structure based on cpDNA sequences revealed two well-differentiated groups of haplotypes. One of them is restricted to the main center of the distribution range in the Tatra Mountains (Mts), while the second group included a series of closely related haplotypes, which in most cases were unique for particular isolated groups of populations in peripheral mountain ranges and in the south-eastern part of the Tatra Mts. AFLP fingerprinting also revealed a pattern of divergence among populations, while only partly corroborating the division observed in cpDNA. Taking into account all the data, the pattern of genetic structure, supported by the high levels of unique genetic markers in populations, may reflect the historical isolation of populations in several local refugia during the last glacial period. Not only the center of the range in the Tatra Mts, but also other, neighboring massifs (Malá Fatra, Nízke Tatry, Chočské vrchy, Muránska planina), where populations are characterized by separate plastid DNA haplotypes, could have acted as separate refugia.

## ﻿Introduction

Mountainous systems of temperate Europe are biodiversity hotspots, due, among others, to their high floristic richness, including endemic species that contribute to the natural uniqueness of a given biogeographical unit. The evolution of mountain species is strongly related to historical environmental and climatic factors, including the Pleistocene climatic fluctuations that caused alternating glacial and interglacial periods (Hewitt 2000, 2004; Kadereit et al. 2004). Phylogeographic analysis of various mountain species allowed us to discover genetic patterns and, to some extent, understand mechanisms and dynamics of processes involved in the formation of local floristic hotspots within large mountain massifs. The results have made it possible to determine the scenarios of events and processes related to the survival of species during glaciations, including the location of refugial areas and recolonization routes to the areas currently occupied by these species. The revealed convergence of genetic diversity patterns between mountain species indicates that similar factors had a prevailing role in shaping the history of the flora in different areas, especially with regard to the history of Quaternary glaciations (e.g., Koch et al. 2003; Kropf et al. 2008; Lowe and Allendorf 2010; Ronikier 2011; Schmitt 2017; Šrámková-Fuxová et al. 2017; Konečná et al. 2019).

Endemic species are a special element of the biodiversity of mountain areas. In this group, species with highly limited local ranges are of particular importance, as they most often reflect evolution in specific microclimatic isolation in geographically small and isolated areas (Kochjarová et al. 2006; Cieślak et al. 2007, 2021; Pittet et al. 2020). Their emergence confirms the role of local ecological niche systems (including topography, climate, and bedrock factors) in shaping the regional mountain flora during historical evolutionary processes. In mountain ranges, establishing an understanding of such local systems is crucial to understanding their contemporary distribution and biodiversity. Consequently, it will allow the determination of the response range of these unique, often isolated, local habitat systems to past and ongoing climate fluctuations, such as the Pleistocene glacial/interglacial sequences (Nagy et al. 2003; Stehlik 2003; Kadereit et al. 2004; Tribsch 2004; Schmitt 2009; Gentili et al. 2015).

It has been confirmed that the Western Carpathians are an important, independent center of endemism with both Pan-Carpathian species and a group of endemic species specific to this region (Pawłowski 1970; Kliment 1999; Piękoś-Mirkowa and Mirek 2003; Mráz and Ronikier 2016). Their location close to the northern ice sheet during glacial periods, heterogeneous environments constituting a mosaic of habitats, and microtopographically diverse environments on a regional scale were crucial to the establishment of an important evolutionary center in this region of the Carpathians (Paclová 1977). Recently, phylogeographic analyses of *Cochleariatatrae*, the endemic species of the Tatra Mts, showed a significant level of intra-specific variability with several geographically arranged genetic groups in this small mountain range (Cieślak et al. 2021). This pattern may reflect the isolation of the populations in several micro-refugia, indicating that the systems of local factors in the Tatra Mts and its outskirts were crucial in formation of the genetic structure of this species.

In this study, we address *Saxifragawahlenbergii* Ball, one of the most characteristic endemic species of the Western Carpathians, with wide ecological preferences, including a large elevational range and limited bedrock restriction. It occurs in several massifs with its range center in the Tatra Mts (Piękoś-Mirkowa et al. 1996). Recent phylogenetic study based on nrDNA and cpDNA regions revealed a complex hybrid origin of *S.wahlenbergii* with unidirectional introgression and different parental contributions observed in extant genotypes (Tkach et al. 2019). The maternal parent has been shown to belong to the West Eurasian lineage of alpine taxa grouped in the subsection Androsaceae, most likely the widespread *S.androsacea*. The putative paternal parent was probably *S.adscendens*, which belongs to a distantly related subsection Tridactylites. Contribution from both groups was confirmed by a next-generation sequencing (NGS) analysis of within-individual ITS variation (Tkach et al. 2019). The supported topological incongruencies between phylogenies reconstructed from nuclear and plastid DNA regions, as previously found (Tkach et al. 2015; Gerschwitz-Eidt and Kadereit 2020), may suggest that interspecific transfer of adaptive traits through hybridization may have played an important role in the evolution of Saxifragasect.Saxifraga. In general, hybrid speciation events involving polyploidization are common in the genus *Saxifraga* and have played an important role in the diversification of this large genus (e.g., Ebersbach et al. 2020).

Interestingly, in the framework of the phylogenetic analysis, regional genetic variation was found in the populations of *S.wahlenbergii* including two distinct cpDNA clades, which was a direct motivation to undertake a more detailed analysis of the species’ phylogeographical structure. While unequivocally dating the hybrid origin of *S.wahlenbergii* could not be assessed, it could theoretically precede the Pleistocene and several arguments pointed to a possibly ancient rather than recent age of this species (Tkach et al. 2019), hence also its geographical range.

In the region of the Western Carpathians, with its high topographic and habitat heterogeneity, *S.wahlenbergii*, a plant with a rather wide altitudinal range, could survive in the massifs where it occurs today, over the Pleistocene climatic oscillations, following the altitudinal shifts or persistence in long-term, non-glaciated microrefugia. However, as cold (glacial) periods have led to a significant increase in habitats suitable for alpine plants in the lower parts of the Carpathians (Ronikier 2011), the present range could also result from a recent (Last Glacial Maximum) migration to peripheral massifs.

The main objective of this study is to determine the range-wide genetic structure of *S.wahlenbergii*, to provide insight into its glacial history. For this purpose, an extended sequence analysis of selected nuclear and plastid DNA regions complementing data from Tkach et al. (2019) and supplemented by population genotyping with Amplified Fragment Length Polymorphism method (Vos et al. 1995; Kirschner et al. 2021), were used. The study was based on the analysis of *S.wahlenbergii* populations from the area of their highest density in the Tatra Mts and those from all neighboring mountain massifs, where it occurs less abundantly (sometimes as isolated populations). Genetic diversity and divergence were analyzed to identify potential distinct lineages and areas of genetic discontinuities of species.

Based on the above data, an attempt was made to resolve whether the contemporary distribution results from long-term survival in several isolated areas (local refugia) and thus is a relic of ancient events or whether the species recently spread from a single refugium (likely located in the Tatra Mts – central part of the range).

## ﻿Materials and methods

### ﻿Study species

*Saxifragawahlenbergii* Ball (sect. Saxifraga; Saxifragaceae) is an endemic perennial species of the Western Carpathians (in Poland and Slovakia). Its range includes the massifs of Tatra Mts, Malá Fatra Mts, Chočské vrchy Mts, Nízke Tatry Mts, and Muránska planina. However, it is a common species only in the Tatra Mts (the highest and environmentally most complex massif of the Western Carpathians) – it is abundant at higher altitudes above the tree line (up to 2540 m a.s.l.) and also descends to lower elevations, e.g., along streams (880 m a.s.l.) (Pawłowska 1966). The species grows on both limestone and granitic substrates, with preference for limestones. Throughout its range, it is found mainly on moist edges of limestone and granite screes, in the shade of rocks, on ledges and in rock crevices or on the edge of forests. It is a characteristic species of the *Saxifragetumwahlenbergii* community (Matuszkiewicz 2005) (described as *Saxifragetumperdurantis*Pawłowski and Stecki 1927). This community is considered an endemic community of the limestone Tatra Mts, Malá Fatra Mts and Chočské vrchy Mts (Matuszkiewicz 2005). Species is a hexaploid with a chromosome number of 2n = 66 (x = 11) (Skalińska 1963).

### ﻿Population sampling

**Figure 1.**
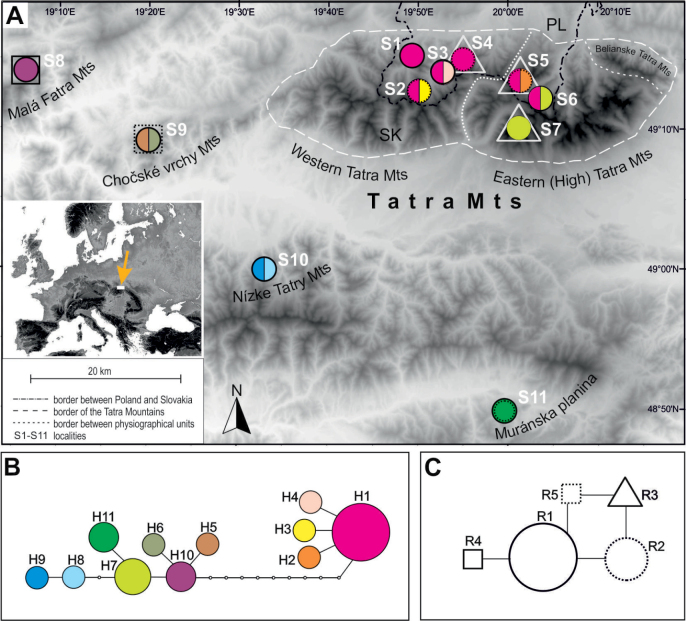
Location of studied populations of *Saxifragawahlenbergii* and their genetic variability based on DNA sequence data **A** distribution of 11 populations of *S.wahlenbergii* and haplotypes and ribotypes in the populations **B** haplotype network based on the combined chloroplast regions: *rps*16-*trn*K and *rpl*32-*trn*L **C** ribotype network based on ITS region. Networks obtained from TCS based on a 95% connection limit. The relative sizes of circles in networks are proportional to haplotype and ribotype frequencies. For population acronyms see Table 1.

Plant material of *Saxifragawahlenbergii* was sampled in natural populations, spanning the entire natural distribution area of this species in the Western Carpathians. Populations were assigned to regional geographical units, which were further assigned into predefined groups: the Western Tatra Mts, the Eastern Tatra Mts and those outside of the Tatra Mts, including localities from: the Malá Fatra Mts, Chočské vrchy Mts, Nízke Tatry Mts and Muránska planina (Kondracki 1989) (Fig. 1A and Table 1 for location details). The number of samples per population varied and depended on population size. Special attention was paid to include samples from all isolated massifs where the species occurs. In total, our dataset comprised 57 individuals collected from 11 populations of *S.wahlenbergii*. Leaves from each individual were placed in a tube or bag with silica gel immediately after collecting and stored at room temperature until the DNA isolation. Herbarium material (vouchers) was collected only from large populations, due to conservation reasons and deposited in the Herbarium of W. Szafer Institute of Botany, Polish Academy of Sciences in Kraków (KRAM).

**Table 1.** Localities of populations of *Saxifragawahlenbergii* used in the study and parameters of their genetic variability based on AFLP, nrDNA (ITS) and cpDNA sequences. N_A_/N_S_ – population sampling for AFLP analysis and DNA sequencing; *P*/% – number and percentage of polymorphic markers; *H*e – mean (±SD) Nei’s gene diversity; *I* – mean (±SD) Shannon’s Index; *DW* – frequency down-weighted marker values; R – ribotypes (variants of ITS of nrDNA) and H – haplotypes (variants of cpDNA) in population (the number of individuals representing a particular ribotype or haplotype is given in parentheses). Country code: PL – Poland, SK – Slovakia. Collectors code: AD – Anna Delimat, AR – Anna Ronikier, MR – Michał Ronikier, RL – Roman Letz, PM – Patrik Mráz, PT – Peter Turis.

Code	Locality	N_A_/N_S_	AFLP				ITS	cpDNA
			*P*/%	*H*e	*I*	*DW*	R(No.)	H(No.)
Western Tatra Mts (Tatry Zachodnie, Západné Tatry)								
S1	PL, Dolina Chochołowska valley, 1370 m a.s.l., 49°14'N, 19°48'E (AD)	5/2	59/27.31	0.10 (±0.18)	0.15 (±0.26)	21.77	R1(2)	H1(2)
S2	PL, between the Gaborowa Przełęcz pass and Bystra Przełęcz pass, ~1930 m a.s.l., 49°12'N, 19°49'E (RL, PM)	4/2	61/28.24	0.12 (±0.19)	0.17 (±0.28)	20.06	R2(2)	H1(1) H3(1)
S3	PL, Przełęcz pod Kopą Kondracką pass, 1500 m a.s.l., 49°14'N, 19°55'E (AD)	5/2	61/28.24	0.11 (±0.18)	0.16 (±0.26)	25.07	R1(2)	H1(1) H4(1)
S4	PL, Piekiełko (Piekło) valley, 1640 m a.s.l., 49°14'N, 19°56'E (AD)	9/2	91/42.10	0.15 (±0.20)	0.23 (±0.28)	58.57	R2(1) R3(1)	H1(2)
Eastern Tatra Mts (High Tatra Mts, Tatry Wysokie, Vysoké Tatry)								
S5	PL, N slopes of the pass Zawrat, 2100 m a.s.l., 49°13'N, 20°01'E (MR)	4/2	79/36.57	0.14 (±0.19)	0.20 (±0.28)	25.46	R2(1) R3(1)	H1(1) H2(1)
S6	PL, Mięguszowiecki Szczyt Czarny Mt., 2220 m a.l.s., 49°11'N, 20°03'E (AD)	7/2	83/38.43	0.14 (±0.20)	0.20 (±0.28)	36.37	R1(2)	H1(1) H7(1)
S7	SK, Hrubý vrch Mt, ~ 2350 m a.s.l., 49°10'N, 20°01'E (MR)	8/2	85/39.35	0.14 (±0.19)	0.21 (±0.28)	40.09	R3(2)	H7(2)
Malá Fatra Mts								
S8	SK, Veľký Rozsutec Mt., 1550 m a.s.l., 49°14'N, 19°06'E (MR, AR)	5/2	76/35.19	0.13 (±0.19)	0.19 (±0.27)	26.90	R1(1) R5(1)	H10 (2)
Chočské vrchy Mts								
S9	SK, Veľký Choč Mt., 1600 m a.s.l., 49°09'N, 19°20'E (MR)	4/2	60/27.78	0.10 (±0.18)	0.15 (±0.26)	23.36	R1(1) R4(1)	H5(1) H6(1)
Nízke Tatry Mts								
S10	SK, Siná Mt., 1422 m a.s.l., 49°00'N, 19°33'E (RL, PT)	4/2	61/28.24	0.11 (±0.19)	0.27 (±0.27)	29.54	R1(2)	H8(1) H9(1)
Spišsko-gemerský kras								
S11	SK, Muránska planina, Vel’ka Stožka, 1242 m a.s.l., 48°46'N, 19°58'E (PT)	–/2	–	–	–	–	R1(1) R2(1)	H11 (2)

### ﻿Laboratory analysis

The total genomic DNA was isolated from 5–10 mg of dried leaf tissue of collected samples using the DNeasy Plant Mini Kit system (Qiagen, Hilden, Germany) according to the manufacturer’s protocol (final elution step was carried out using 2×50 μL of elution buffer). DNA quality and concentration were estimated against λ-DNA on 1% agarose gel stained with ethidium bromide. The purified DNA isolates were the basis of DNA sequencing and AFLP analyses. Samples from the Muránska planina population (S11) were collected later than the core sample set and they could only be used in the sequencing analysis.

The non-coding chloroplast DNA regions (cpDNA) – *rps*16-*trn*K and *rpl*32-*trn*L (Shaw et al. 2007) – and nuclear ribosomal DNA region (nrDNA) – ITS (*ITS*1A-*ITS*4, White et al. 1990; Blattner 1999) – were used for DNA sequencing. Primers were selected and analyzed according to the protocols described in detail by Tkach et al. (2015, 2019).

AFLP analysis was performed according to Vos et al. (1995) as described in detail by Cieślak et al. (2007). For high-quality AFLP profiles, we tested selective primer combinations using four individuals from distant populations. All samples were analyzed using three selective primers combinations that yielded clear, unambiguous, and polymorphic profiles – *EcoRI*-AAG/*MseI*-CTG, *EcoRI*-ACT/*MseI*-CAG and *EcoRI*-AGA/*MseI*-CAC. Genotyping reproducibility was tested by including duplicates for each population (Bonin et al. 2004). Amplification products were separated with an internal size standard (GeneScan ROX-500) on the ABI Prism 3100 Avant automated sequencer using POP-4 polymer (Applied Biosystems, Foster City, CA, USA). Obtained AFLP marker sets were imported to Genographer Software (v. 1.6.0; J. Benham, Montana State University, 1998–2001) (Benham et al. 1999), which was used to score the fragments in the range of 50–500 bp. AFLP fragments for primer combination were saved as present (1) or absent (0) for a binary data matrix (see Suppl. material 1).

### ﻿cpDNA and ITS of nrDNA data analysis

Analyses of cpDNA and ITS of nrDNA regions were performed separately. Forward and reverse DNA sequences data were automatically assembled and aligned based on ClustalW algorithm (Thompson et al. 1994; Larkin et al. 2007) using the Geneious Pro 6.0.2 program (Drummond et al. 2011). The obtained sequences are deposited in GenBank with accession numbers: *rps*16-*trn*K – OQ706232–53; *rpl*32-*trn*L – OQ706254–73, OR682717–18 and *ITS*1A-*ITS*4 – OQ678158–79 (see Suppl. material 2).

Gene diversity (*h*) and nucleotide diversity (π) were calculated based on cpDNA and ITS sequence variation for the total sample of *Saxifragawahlenbergii* and for predefined region groups within the species range using the DNAsp 5.0 program (Librado and Rozas 2009). To detect past demographic expansion, evidence for possible selection and/or genetic bottlenecks, Tajima’s *D* neutrality test was implemented (Tajima 1989). Indels were treated as single polymorphic sites. The relationships between nrDNA ITS ribotypes (R) and cpDNA haplotypes (H) were separately analyzed using the statistical parsimony (SP) algorithm (Templeton et al. 1992) as implemented in TCS v1.2 (Clement et al. 2000); coding indels longer than 1 bp were treated as single characters. Statistical parsimony networks and the maximum number of mutational steps were obtained with 95% connection limit approach. Phylogenetic reconstruction using Bayesian inference was accomplished with MrBayes 3.2.7a program (Ronquist et al. 2012). The analysis was completed for four chains, parameter values of nst=6 and rates=invgamma, 50 million generations with sampling trees every 100 generations. 25% of the initial trees were discarded and the remaining 75% were used to build majority consensus tree and to calculate Bayesian posterior probabilities. The tree was visualized using FigTree 1.4.2 (Rambaut 2014).

### ﻿AFLP data analysis

The genetic diversity of *Saxifragawahlenbergii* at species and within-species level (populations) was assessed on the basis of binary AFLP data matrix by calculating the genetic parameters, including the number (*P*) and percentage of polymorphic markers (%) , Nei’s gene diversity (*H*e), Shannon’s information index (*I*) and gene flow (*Nm*) using POPGENE v. 1.32 software (Yeh et al. 1999). In order to identify long-term isolated and genetically unique populations, frequency down-weighed marker values (*DW*; Schönswetter and Tribsch 2005) were calculated using R-script AFLPdat (Ehrich 2006). The relationships among individuals and populations were analyzed by a Principal Coordinates Analysis (PCoA) based on the Nei-Li genetic distance matrix computed in FAMD v. 1.25 (Schlüter and Harris 2006) and by a split network (Neighbor-Net) also based on the Nei-Li coefficient with branch support estimated by bootstrapping with 1000 replicates, implemented in SPLITStree4 (Huson and Bryant 2006). Further, the model-based Bayesian clustering procedure in Structure v. 2.3.4 (Pritchard et al. 2000) was used to determine the genetic structure of populations. The analysis was performed by setting the number of populations (*K*) from 2 to 12. The burn-in steps and the number of replicates were 10,000 and 50,000 for each *K*, respectively. All runs were repeated 100 times at each *K* and the optimal *K* value was selected as a point of a marked change in the envelope slope (kink of the curve) of lnP(D) as a function of *K*. Genetic population structure was investigated by a hierarchical analysis of molecular variance (AMOVA), and relationships between populations from different parts of the species’ range were assessed based on pairwise genetic divergence (*F*_ST_) for all populations, both implemented in Arlequin v. 3.5 (Excoffier and Lischer 2010).

## ﻿Results

### ﻿cpDNA and ITS of nrDNA variation

The sequences of cpDNA and ITS of nrDNA regions were obtained from 22 individuals from eleven populations of *Saxifragawahlenbergii* (Table 1).

The alignments of *rps*16-*trn*K and *rpl*32–*trn*L regions of cpDNA were 796 bp and 655 bp in length, respectively (concatenated cpDNA alignment – 1451 bp in length). 15 variable sites were found – 4 singleton variable sites and 11 parsimony informative sites, which represented transitions (C-T, 3 A-G) and transversions (3 G-T, 2 A-T and 2 A-C). In the *rpl*32–*trn*L region a thirty-one-nucleotide insertion/deletion was also identified. Eleven haplotypes (H1–H11; Fig. 1A, B) determined by these polymorphisms were revealed.

**Figure 2.**
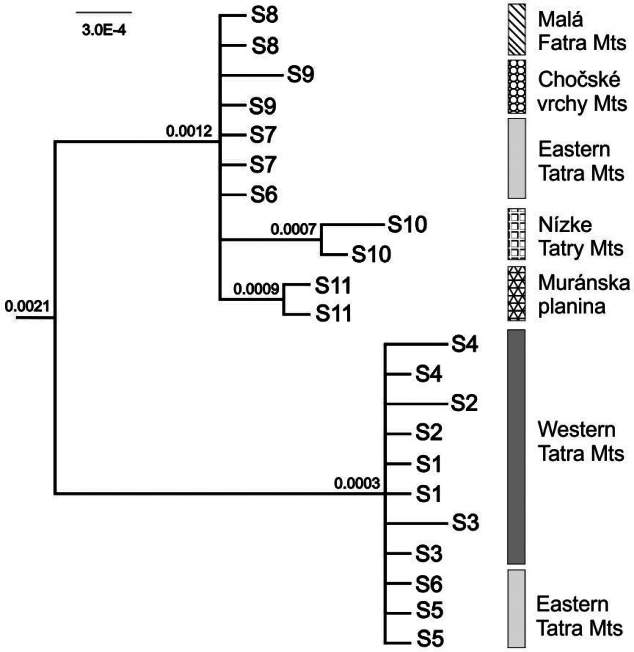
MrBayes tree based on the combined plastid *rps*16–*trn*K and *rpl*32–*trn*L regions of *Saxifragawahlenbergii* (reduced dataset; see text for details). Numbers at nodes, in the order shown, correspond to posterior probabilities estimated in MrBayes. For population acronyms see Table 1.

Each population harbored one or two cpDNA haplotypes, mostly specific for individual populations and/or mountain ranges. H1–H4 and H7 haplotypes occurred only in the Tatra Mts, with the most frequent H1 haplotype present in almost all Tatra populations (six out of seven populations) (Fig. 1A, B, Tables 1, 2). On the other hand, H8–H11 haplotypes were detected exclusively in populations from the isolated locations outside the Tatra Mts. Accordingly, the network of cpDNA haplotypes based on statistical parsimony analysis revealed two main groups separated by nine mutations and corresponding to the Tatra Mts versus other mountain ranges (Fig. 1A, B). In the Tatra Mts group, haplotypes displayed a star-like pattern, with H1 as the dominant haplotype, widespread across the Western Tatra Mts and the Eastern Tatra Mts. In the second group from outside the Tatra Mts, haplotypes occurred with comparable frequency. The phylogenetic tree, as inferred through Bayesian analysis, revealed two distinct groups – individuals from the Tatra Mts form a sister genetic group to the group including all other individuals. A certain level of divergence was observed only among individuals from the Eastern Tatra Mts in both clades (Fig. 2).

**Table 2.** Genetic diversity of nrDNA and cpDNA sequences of *Saxifragawahlenbergii* calculated for a priori delimitation of regional groups. R – ribotypes (variants of nrDNA ITS) and H – haplotypes (variants of cpDNA); *h* – mean (±SD) gene diversity; π – mean (±SD) nucleotide diversity; *D* – Tajima’s *D* statistic value; *non-significant at the 5% level (*P* > 0.05).

Regional groups	ITS				cpDNA			
	R	*h*	π	*D*	H	*h*	π	*D*
Western Tatra Mts	R1, R2, R3	0.00	0.00	0.00	H1, H3, H4	0.46 (±0.20)	0.0003 (±0.0007)	-1.31*
Eastern Tatra Mts	R1, R2, R3	0.00	0.00	0.00	H1, H2, H7	0.73 (±0.16)	0.0036 (±0.0004)	1.80*
Tatra Mts (as a whole)	R1, R2, R3	0.00	0.00	0.00	H1, H2, H3, H4, H7	0.66 (±0.12)	0.0024 (±0.0008)	-0.15*
Outside of the Tatra Mts	R1, R2, R4, R5	0.00	0.00	0.00	H5, H6, H8, H9, H10, H11	0.86 (±0.11)	0.0013 (±0.0003)	-0.33*

Overall, *S.wahlenbergii* displays a moderate gene diversity (*h* = 0.81 ±0.06) and nucleotide diversity (π = 0.0036 ±0.0003) of cpDNA. At the local level of predefined regional groups, the highest gene diversity (*h* = 0.86 ±0.11) was found in the group of populations outside of the Tatra Mts (i.e., in Malá Fatra Mts, Chočské vrchy Mts, Nízke Tatry Mts and Muránska planina) with low nucleotide diversity (π = 0.0013 ±0.0003; Table 2). Within the Tatra Mts, populations of *S.wahlenbergii* from the eastern part were characterized by higher gene (*h* = 0.73 ±0.16) and nucleotide diversity (π = 0.0036 ±0.0004) than populations from the western part of this mountain range (*h* = 0.46 ±0.20; π = 0.0003 ±0.0007, respectively). Testing deviation from neutrality (Tajima’s *D*) revealed no significant indications for departure from neutrality within regions (*P* > 0.05).

The obtained ITS alignment was 730 bp long, with very low sequence diversity. Only four indels (three poly-A and one poly-G stretches) were found and on this basis five ribotypes were established (R1–R5) (Fig. 1A, C). Only R1 ribotype was widespread and shared by seven populations within the species’ range. The following ribotypes were specific for geographical regions: R3 for the Tatra Mts, R4 for the Malá Fatra Mts and R5 for the Chočské vrchy Mts (Tables 1, 2). The network analysis indicated close links between five ribotypes, and internal divisions into groups (Fig. 1C).

### ﻿AFLP variation

The AFLP analysis yielded 213 DNA markers, of which 181 (84.98%) were polymorphic for 55 individuals from ten populations of *Saxifragawahlenbergii* (Table 1). Reproducibility of obtained AFLP band profiles was ~96%. The number of polymorphic markers in populations ranged from 59 (S1) to 91 (S4), with a mean of 83 markers (±20.29) per individual. There were no identical AFLP phenotypes among studied individuals. Only in populations from the Veľký Choč Mt. (S9, Chočské vrchy Mts) and Siná Mt. (S10, Nízke Tatry Mts) one private marker in each was identiﬁed.

At the species level, Nei’s gene diversity (*H*e) was 0.16 (±0.17) and ranged from 0.10 (S1) to 0.15 (S4), with an average value of 0.12 (±0.02). The frequency of down-weighted markers (*DW*) was similar across most populations and ranged from 20 to 30, with much higher values in populations S4 (59), S7 (40) and S6 (36) (Table 1).

**Figure 3.**
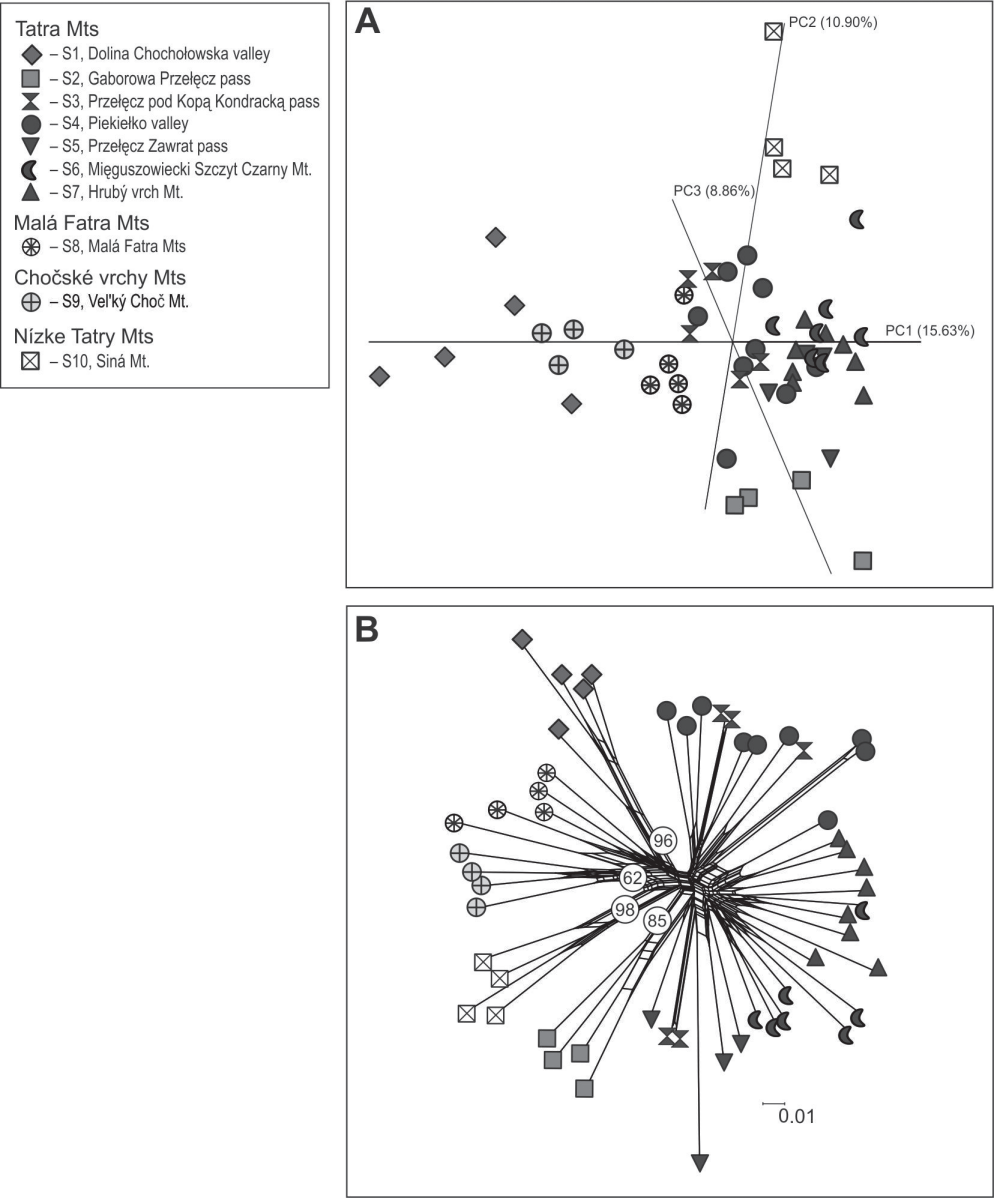
Phylogeographic structure within of *Saxifragawahlenbergii* based on AFLP dataset (55 individuals from 10 populations) **A** Principal Component Analysis diagram, ordination at 1 *vs* 2 *vs* 3 axes **B** Neighbor-Net diagram with the bootstrap values derived from an analysis of 2,000 replicates above 50% has been given. Both diagrams were prepared based on the Nei-Li coefficient. For population acronyms see Table 1.

The further analysis of PCoA performed on the entire dataset revealed that *S.wahlenbergii* populations are not clearly genetically divergent and form partially overlapping groups. In general, the population’s scatter is characterized by the west-east gradient across the distribution range of *S.wahlenbergii*. In 1–3 axes arrangement, the populations from the disjunct parts of the range (populations: S1, S8, S9 and S10) are opposite to the highest locations of Hrubý vrch Mt. and Mięguszowiecki Szczyt Czarny Mt. (the Eastern Tatra Mts). In the central part of plot, individuals from population of the Western Tatra Mts (S3, S4) and Malá Fatra Mts were located. The first three factors of the PCoA accounted for 35.39% of the total variation in the dataset (Fig. 3A).

The Neighbor-Net diagram demonstrated two groups, each consisting of clusters representing single, spatially isolated populations, but with different bootstrap support. The first group included those with higher bootstrap values, such as Nízke Tatry Mts (98%), Dolina Chochołowska valley (96%), Gaborowa Przełęcz pass (85%), Veľký Choč Mt. (62%) and Malá Fatra Mts (61%). The second group consisted of populations with very low support (Fig. 3B). It is characteristic that individuals of *S.wahlenbergii* from the higher altitudes of the Eastern Tatra Mts were closely related with each other and genetically more distant from individuals from slightly lower altitudes in the Western Tatra Mts.

Analysis of genetic variation (AMOVA) showed that a major part of *S.wahlenbergii* variation is attributed to the within-population level – 72.09%, in relation to among-population variation – 27.91% (*F*_ST_ = 0.28, *P* < 0.001). The same pattern can be found when analyzing geographical groups (Table 3, see Suppl. material 3). AMOVA also confirmed low but statistically significant genetic differences between regions (*F*_CT_ = 0.05, *P* < 0.01). Based on the comparison of *F*_ST_ values between pairs of populations, a gradation in differentiation between the populations of *S.wahlenbergii* from individual range regions was found. The population from Nízke Tatry Mts is the most distinct one, while the population from Malá Fatra Mts shows greater similarity with those from the Tatra Mts compared to the other parts of the range (see Suppl. materials 4, 5). Within the Tatra Mts, populations from the Eastern Tatra Mts presented greater genetic affinity with each other than with populations from the Western Tatra Mts. The greater mean diversity of the population in the Western Tatra Mts is due to the significant distinctiveness of the population from the Dolina Chochołowska valley, where the *F*_ST_ values between other populations range from 0.36 to 0.49 (see Suppl. material 4).

**Table 3.** AMOVA analysis based on AFLP data for the populations of *Saxifragawahlenbergii* calculated for all populations and a priori delimitation of regional groups. Significance tests based on 1023 permutations, ***P < 0.001, **P < 0.01.

Source of variation	d.f.	Sums of Squares	Variance components	% Total variance	*F* statistics
Among populations	9	487.480	6.753	27.91***	*F*_ST_ = 0.28
Within populations	45	784.738	17.439	72.09	
Total	54	1272.218	24.192		
Among regional groups – Western Tatra Mts*vs.* Eastern (High) Tatra Mts*vs.* outside of the Tatra Mts	2	145.588	1.206	4.92**	*F*_CT_ = 0.05
Among populations	7	341.892	5.872	23.95***	*F*_SC_ = 0.25
Within population	45	784.738	17.439	71.13	*F*_ST_ = 0.29
Total	54	1272.218	24.517		

**Figure 4.**
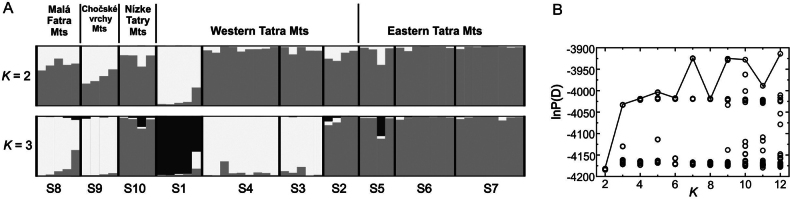
**A** The histograms representing the assignment of 55 individuals of *Saxifragawahlenbergii* to different clusters by Bayesian spatial clustering (STRUCTURE software). Each vertical bar corresponds to an individual, highlighted in gray for clarity, contrasting with the cluster assignments, respectively, at *K* = 2 and *K* = 3 **B** in P(Data) values in function of *K* are shown. For population acronyms see Table 1.

AFLP data indicates a low level of gene flow between populations of *S.wahlenbergii* (*Nm* = 0.66). In the Structure analysis of AFLP data, the stable and optimal number of population groups was selected based on the kink in the envelope of lnP(D) values. As can be seen from Fig. 4A, B, a clear change in the slope was found for *K* = 3. In order to further justify the reasons for such a choice, similar analyses were performed both for *K* = 2 and 3. For *K* = 2, the population from Dolina Chochołowska valley, represented a nearly homogeneous and clearly separated genetic group whereas populations from Eastern Tatra Mts, Nízke Tatry Mts and remaining populations from the Western Tatra Mts formed the second one. The populations from Malá Fatra Mts and Veľký Choč Mts displayed genetic admixture with a significant contribution of both genetic pools. For *K* = 3, population from the Dolina Chochołowska valley is even more genetically distinct, while all the populations from the Eastern Tatra Mts, Gaborowa Przełęcz pass in the Western Tatra Mts and Nízke Tatry Mts are similar to each other. Remaining populations from the Western Tatra Mts, as well as from Malá Fatra Mts and Veľký Choč Mts, represent the third group.

## ﻿Discussion

Phylogenetic analyses of the Saxifraga section indicated the hybrid origin of *Saxifragawahlenbergii* and allowed us to estimate the possible oldest age of its hybrid origin to the late Neogene (4.7 Ma). These analyses also provided insights into its internal diversity (Tkach et al. 2019). Our analysis confirmed two distinct groups of plastid haplotypes corresponding to the geographical locations of populations and revealed additional local phylogeographical structures. This distribution of genetic lineages on a small spatial scale, characteristic of the Tatra Mts, was also observed in the endemic species *Cochleariatatrae* (Koch et al. 2003; Cieślak et al. 2021). This spatial pattern, along with the presence of unique haplotypes in populations, implies survival in local refugia with limited contact among them.

In mountain conditions, the process of isolation by distance contributed to historical interruption of gene flow between populations, leading to geographically driven groups of populations (e.g., Tribsch and Schönswetter 2003; Schönswetter et al. 2005; Kropf et al. 2006; Christe et al. 2014; Melichárková et al. 2019). Consequently, contemporary populations represent more or less distinct units in the landscape, potentially facilitated by the periglacial environment. This environment consisted of a mosaic of habitats that allowed the survival of various population groups (Birks and Willis 2008; Provan and Bennett 2008). The accumulation of intraspecific diversity in *S.wahlenbergii*, particularly within the cpDNA, among populations from different regions, such as the Malá Fatra Mts, Chočské vrchy Mts, Nízke Tatry Mts, and Muránska planina, points to the scenario that these regions may have acted as distinct glacial refugia for high mountain species in the Western Carpathians. Analyses of AFLP data highlight a weakly resolved but distinct position of spatially isolated populations and groups, further supporting the above interpretation.

In the Tatra Mts, subalpine populations of *S.wahlenbergii* are more closely related to those geographically closest from the same mountain range than to their subalpine counterparts from other mountain ranges. This suggests that the source area of their recolonization could have been populations from low elevations, such as the extant population from the Dolina Chochołowska valley, a site which remained outside the glaciation area (Kłapyta et al. 2016; Kłapyta and Zasadni 2017–2018). In the Tatra Mts, which are clearly different from their periphery, a high genetic diversity of haplotypes is observed. Apart from several haplotypes from both cpDNA groups, the presence of R2 and R3 ITS variants is restricted to populations from the Tatras range. The genetic structure of *S.wahlenbergii* in this area was likely influenced by the topographically complex environment and historical conditions in the glacial periods.

The Tatra Mts, unlike most of the Western Carpathians, were strongly, albeit unevenly, glaciated during the Pleistocene glaciations (Zasadni and Kłapyta 2014; Zasadni et al. 2022). Due to the occurrence of extensive glaciers in the valleys (Kłapyta et al. 2016), available local glacial refugia were physically isolated. Within the mountains, refugia were generally distributed along steep, uncovered rocky crests at the highest altitudes and lower crests below the snowline. Large areas with a mosaic of habitats potentially suitable for high-mountain plants were also available in adjacent low-altitude locations along the entire range. Consequently, survival in lower, periglacial habitats appears appropriate for *S.wahlenbergii*. During cold glacial periods, these could have been places both at the foot and along the glacial moraines, in the glacier ablation zones, as well as in the areas occupied by steppe-tundra. Survival in these areas could have been possible thanks to the ability of species to live in a wide range of habitat conditions, such as moist edges of limestone and granite scree, in the shade of rocks, on shelves and in rock crevices.

On the other hand, low values of *F*_ST_ (the lowest in relation to the compared pairs of populations) observed in the AFLP data from the Tatra Mts can be the result of the maintenance of gene ﬂow between populations during recolonization of this area after the last glaciation and may counteract incipient differentiation processes, thereby avoiding bottlenecks, genetic drift, and the loss of genetic diversity. Characteristically, the highest values of the *F*_ST_ were noted between populations from the areas with the highest altitudes, namely the Tatra Mts and the Nízke Tatry Mts. These results suggest that mountain ridges acted as a stronger barrier for gene flow more effectively than the elevation differences between subalpine and lower-lying areas within the same ranges. In addition, the genetic structure of *S.wahlenbergii*, a relic mountain plant species, certainly reflects processes acting in different time periods. Populations that survived when environmental conditions became unfavorable could retain genetic variability. Becoming a source of remigration in new conditions, they could also host new local mutation fixations. Therefore, it can be assumed that both Quaternary climatic oscillations and ecological divergence have played a role in shaping the distribution and divergence patterns observed in *S.wahlenbergii*. Similarly, in the species complex *Alyssum montanum–A. repens*, a clear elevational shift was identified, indicating that differential ecological adaptation occurred in the respective mountain areas (Melichárková et al. 2019). It should be emphasized that these findings are consistent with previous results of phylogeographical analyses (Ronikier et al. 2012; Cieślak et al. 2021), which showed the Tatra Mountains as an important, independent area within the Western Carpathians, where the local structure of species was formed. These patterns indicate that the Tatra Mts also served as a refugium or a system of microrefugia, likely due to their high topographical and habitat diversity. However, the genetic position of populations from remaining massifs, especially the series of massif-specific cpDNA haplotypes, also indicates their role supporting the *S.wahlenbergii* populations, forming a system of discrete parts of the range likely over a longer temporal scale. In the case of an allopolyploid species of hybrid origin it cannot also be ruled out that a polytopic hybrid origin may have played a role in the two main intraspecific lineages observed (Melichárková et al. 2019), which have later undergone an internal diversification in the isolated mountain environment.

## ﻿Additional information

### Author ORCIDs

Elżbieta Cieślak https://orcid.org/0000-0002-6267-9333

Michał Ronikier https://orcid.org/0000-0001-7652-6787

Magdalena Szczepaniak https://orcid.org/0000-0002-7483-3932

### Data availability

All of the data that support the findings of this study are available in the main text or Supplementary Information.

## Supplementary materials

Supplementary material 1 AFLP dataset of *Saxifragawahlenbergii* Data type xls https://binary.pensoft.net/file/1136864This dataset is made available under the Open Database License (http://opendatacommons.org/licenses/odbl/1.0/). The Open Database License (ODbL) is a license agreement intended to allow users to freely share, modify, and use this Dataset while maintaining this same freedom for others, provided that the original source and author(s) are credited.Elżbieta Cieślak, Michał Ronikier, Magdalena Szczepaniak

Supplementary material 2 The datasets of nrDNA and cpDNA of *Saxifragawahlenbergii* Data type docx https://binary.pensoft.net/file/1136865This dataset is made available under the Open Database License (http://opendatacommons.org/licenses/odbl/1.0/). The Open Database License (ODbL) is a license agreement intended to allow users to freely share, modify, and use this Dataset while maintaining this same freedom for others, provided that the original source and author(s) are credited.Elżbieta Cieślak, Michał Ronikier, Magdalena Szczepaniak

Supplementary material 3 AMOVA analysis based on AFLP data for populations of *Saxifragawahlenbergii* calculated with a priori delimitation of regional groups Data type docx Explanation note Significance tests based on 1023 permutations; **P* < 0.001. Regional grouping of populations see in Materials and methods. https://binary.pensoft.net/file/1136866This dataset is made available under the Open Database License (http://opendatacommons.org/licenses/odbl/1.0/). The Open Database License (ODbL) is a license agreement intended to allow users to freely share, modify, and use this Dataset while maintaining this same freedom for others, provided that the original source and author(s) are credited.Elżbieta Cieślak, Michał Ronikier, Magdalena Szczepaniak

Supplementary material 4 Pairwise genetic divergence (*F*_ST_) across 10 populations of *Saxifragawahlenbergii* based on AFLP data Data type docx Explanation note Significance tests based on 1023 permutations; *P* < 0.001. For population acronyms see Table 1. https://binary.pensoft.net/file/1136867This dataset is made available under the Open Database License (http://opendatacommons.org/licenses/odbl/1.0/). The Open Database License (ODbL) is a license agreement intended to allow users to freely share, modify, and use this Dataset while maintaining this same freedom for others, provided that the original source and author(s) are credited.Elżbieta Cieślak, Michał Ronikier, Magdalena Szczepaniak

Supplementary material 5 Spatial arrangement of varying divergences (*F*_ST_) among populations within distribution range of *Saxifragawahlenbergii* Data type pdf Explanation note Above the line, the average *F*_ST_ values are given; the line colors correspond to scale in the right corner of the map. For population acronyms see Table 1. https://binary.pensoft.net/file/1136868This dataset is made available under the Open Database License (http://opendatacommons.org/licenses/odbl/1.0/). The Open Database License (ODbL) is a license agreement intended to allow users to freely share, modify, and use this Dataset while maintaining this same freedom for others, provided that the original source and author(s) are credited.Elżbieta Cieślak, Michał Ronikier, Magdalena Szczepaniak
