# Supplementary material for: ﻿Glacial history of Saxifragawahlenbergii (Saxifragaceae) in the context of refugial areas in the Western Carpathians
Source: PhytoKeys. 2024 Sep 20;246:295–314. doi: 10.3897/phytokeys.246.118796 (PMC11437128; doi:10.3897/phytokeys.246.118796)
Supplement: Supplementary material 2 — The datasets of nrDNA and cpDNA of Saxifragawahlenbergii [file phytokeys-246-295_article-118796__-s002.docx]

The datasets of nrDNA and cpDNA presented in this study can be found in online repositories in Genbank ([www.ncbi.nlm.nih.gov/Genbank](http://www.ncbi.nlm.nih.gov/Genbank)). The names of the repository/repositories and accession number(s) can be found below:

OQ706232 - <https://www.ncbi.nlm.nih.gov/nuccore/OQ706232>

OQ706233 - <https://www.ncbi.nlm.nih.gov/nuccore/OQ706233>

OQ706234 - <https://www.ncbi.nlm.nih.gov/nuccore/OQ706234>

OQ706235 - <https://www.ncbi.nlm.nih.gov/nuccore/OQ706235>

OQ706236 - <https://www.ncbi.nlm.nih.gov/nuccore/OQ706236>

OQ706237 - <https://www.ncbi.nlm.nih.gov/nuccore/OQ706237>

OQ706238 - <https://www.ncbi.nlm.nih.gov/nuccore/OQ706238>

OQ706239 - <https://www.ncbi.nlm.nih.gov/nuccore/OQ706239>

OQ706240 - <https://www.ncbi.nlm.nih.gov/nuccore/OQ706240>

OQ706241 - <https://www.ncbi.nlm.nih.gov/nuccore/OQ706241>

OQ706242 - <https://www.ncbi.nlm.nih.gov/nuccore/OQ706242>

OQ706243 - <https://www.ncbi.nlm.nih.gov/nuccore/OQ706243>

OQ706244 - <https://www.ncbi.nlm.nih.gov/nuccore/OQ706244>

OQ706245 - <https://www.ncbi.nlm.nih.gov/nuccore/OQ706245>

OQ706246 - <https://www.ncbi.nlm.nih.gov/nuccore/OQ706246>

OQ706247 - <https://www.ncbi.nlm.nih.gov/nuccore/OQ706247>

OQ706248 - <https://www.ncbi.nlm.nih.gov/nuccore/OQ706248>

OQ706249 - <https://www.ncbi.nlm.nih.gov/nuccore/OQ706249>

OQ706250 - <https://www.ncbi.nlm.nih.gov/nuccore/OQ706250>

OQ706251 - <https://www.ncbi.nlm.nih.gov/nuccore/OQ706251>

OQ706252 - <https://www.ncbi.nlm.nih.gov/nuccore/OQ706242>

OQ706253 - <https://www.ncbi.nlm.nih.gov/nuccore/OQ706253>

OQ706254 - <https://www.ncbi.nlm.nih.gov/nuccore/OQ706254>

OQ706255 - <https://www.ncbi.nlm.nih.gov/nuccore/OQ706255>

OQ706256 - <https://www.ncbi.nlm.nih.gov/nuccore/OQ706256>

OQ706257 - <https://www.ncbi.nlm.nih.gov/nuccore/OQ706257>

OQ706258 - <https://www.ncbi.nlm.nih.gov/nuccore/OQ706258>

OQ706259 - <https://www.ncbi.nlm.nih.gov/nuccore/OQ706259>

OQ706260 - <https://www.ncbi.nlm.nih.gov/nuccore/OQ706260>

OQ706261 - <https://www.ncbi.nlm.nih.gov/nuccore/OQ706261>

OQ706262 - <https://www.ncbi.nlm.nih.gov/nuccore/OQ706262>

OQ706263 - <https://www.ncbi.nlm.nih.gov/nuccore/OQ706263>

OQ706264 - <https://www.ncbi.nlm.nih.gov/nuccore/OQ706264>

OQ706265 - <https://www.ncbi.nlm.nih.gov/nuccore/OQ706265>

OQ706266 - <https://www.ncbi.nlm.nih.gov/nuccore/OQ706266>

OQ706267 - <https://www.ncbi.nlm.nih.gov/nuccore/OQ706267>

OQ706268 - <https://www.ncbi.nlm.nih.gov/nuccore/OQ706268>

OQ706269 - <https://www.ncbi.nlm.nih.gov/nuccore/OQ706269>

OQ706270 - <https://www.ncbi.nlm.nih.gov/nuccore/OQ706270>

OQ706271 - <https://www.ncbi.nlm.nih.gov/nuccore/OQ706271>

OQ706272 - <https://www.ncbi.nlm.nih.gov/nuccore/OQ706272>

OQ706273 - <https://www.ncbi.nlm.nih.gov/nuccore/OQ706273>

OR682717 - <https://www.ncbi.nlm.nih.gov/nuccore/OR682717>

OR682718 - <https://www.ncbi.nlm.nih.gov/nuccore/OR682718>

OQ678158 - <https://www.ncbi.nlm.nih.gov/nuccore/OQ678158>

OQ678159 - <https://www.ncbi.nlm.nih.gov/nuccore/OQ678159>

OQ678160 - <https://www.ncbi.nlm.nih.gov/nuccore/OQ678160>

OQ678161 - <https://www.ncbi.nlm.nih.gov/nuccore/OQ678161>

OQ678162 - <https://www.ncbi.nlm.nih.gov/nuccore/OQ678162>

OQ678163 - <https://www.ncbi.nlm.nih.gov/nuccore/OQ678163>

OQ678164 - <https://www.ncbi.nlm.nih.gov/nuccore/OQ678164>

OQ678165 - <https://www.ncbi.nlm.nih.gov/nuccore/OQ678165>

OQ678166 - <https://www.ncbi.nlm.nih.gov/nuccore/OQ678166>

OQ678167 - <https://www.ncbi.nlm.nih.gov/nuccore/OQ678167>

OQ678168 - <https://www.ncbi.nlm.nih.gov/nuccore/OQ678168>

OQ678169 - <https://www.ncbi.nlm.nih.gov/nuccore/OQ678169>

OQ678170 - <https://www.ncbi.nlm.nih.gov/nuccore/OQ678170>

OQ678171 - <https://www.ncbi.nlm.nih.gov/nuccore/OQ678171>

OQ678172 - <https://www.ncbi.nlm.nih.gov/nuccore/OQ678172>

OQ678173 - <https://www.ncbi.nlm.nih.gov/nuccore/OQ678173>

OQ678174 - <https://www.ncbi.nlm.nih.gov/nuccore/OQ678174>

OQ678175 - <https://www.ncbi.nlm.nih.gov/nuccore/OQ678175>

OQ678176 - <https://www.ncbi.nlm.nih.gov/nuccore/OQ678176>

OQ678177 - <https://www.ncbi.nlm.nih.gov/nuccore/OQ678177>

OQ678178 - <https://www.ncbi.nlm.nih.gov/nuccore/OQ678178>

OQ678179 - <https://www.ncbi.nlm.nih.gov/nuccore/OQ678179>
