# Supplementary material for: ﻿Glacial history of Saxifragawahlenbergii (Saxifragaceae) in the context of refugial areas in the Western Carpathians
Source: PhytoKeys. 2024 Sep 20;246:295–314. doi: 10.3897/phytokeys.246.118796 (PMC11437128; doi:10.3897/phytokeys.246.118796)
Supplement: Supplementary material 3 — AMOVA analysis based on AFLP data for populations of Saxifragawahlenbergii calculated with a priori delimitation of regional groups [file phytokeys-246-295_article-118796__-s003.docx]

**Table S1.** AMOVA analysis based on AFLP data for populations of *Saxifraga wahlenbergii* calculated with a priori delimitation of regional groups. Significance tests based on 1023 permutations;**P* < 0.001. Regional grouping of populations see in Materials and methods.

| Source of variation | d.f. | Sums of  squares | Variance components | % total  variance | *F* statistics |
| --- | --- | --- | --- | --- | --- |
| Western Tatra Mts |  |  |  |  |  |
| Among populations | 3 | 177.575 | 7.613 | 30.88* | *F*_ST_ = 0.31 |
| Within populations | 19 | 323.817 | 17.043 | 69.12 |  |
| Total | 22 | 501.391 | 24.656 |  |  |
| Eastern (High) Tatra Mts |  |  |  |  |  |
| Among populations | 2 | 60.994 | 2.013 | 9.95* | *F*_ST_ = 0.10 |
| Within populations | 16 | 291.321 | 8.208 | 90.05 |  |
| Total | 18 | 352.316 | 20.221 |  |  |
| Outside of the Tatra Mts |  |  |  |  |  |
| Among populations | 2 | 103.323 | 8.056 | 32.20* | *F*_ST_ = 0.32 |
| Within populations | 10 | 169.600 | 16.960 | 67.80 |  |
| Total | 12 | 272.923 | 25.016 |  |  |
