# Supplementary material for: ﻿Glacial history of Saxifragawahlenbergii (Saxifragaceae) in the context of refugial areas in the Western Carpathians
Source: PhytoKeys. 2024 Sep 20;246:295–314. doi: 10.3897/phytokeys.246.118796 (PMC11437128; doi:10.3897/phytokeys.246.118796)
Supplement: Supplementary material 4 — Pairwise genetic divergence (FST) across 10 populations of Saxifragawahlenbergii based on AFLP data [file phytokeys-246-295_article-118796__-s004.docx]

**Table S2.** Pairwise genetic divergence (*F*_ST_) across 10 populations of *Saxifraga wahlenbergii* based on AFLP data. Significance tests based on 1023 permutations; *P* < 0.001. For population acronyms see Table 1.

|  | S1 | S2 | S3 | S4 | S5 | S6 | S7 | S8 | S9 | S10 |
| --- | --- | --- | --- | --- | --- | --- | --- | --- | --- | --- |
| S1 | 0.00 |  |  |  |  |  |  |  |  |  |
| S2 | 0.45 | 0.00 |  |  |  |  |  |  |  |  |
| S3 | 0.40 | 0.35 | 0.00 |  |  |  |  |  |  |  |
| S4 | 0.36 | 0.30 | 0.07 | 0.00 |  |  |  |  |  |  |
| S5 | 0.36 | 0.23 | 0.15 | 0.14 | 0.00 |  |  |  |  |  |
| S6 | 0.41 | 0.31 | 0.22 | 0.19 | 0.11 | 0.00 |  |  |  |  |
| S7 | 0.41 | 0.27 | 0.25 | 0.14 | 0.11 | 0.08 | 0.00 |  |  |  |
| S8 | 0.37 | 0.30 | 0.26 | 0.20 | 0.20 | 0.23 | 0.23 | 0.00 |  |  |
| S9 | 0.42 | 0.40 | 0.32 | 0.29 | 0.30 | 0.36 | 0.35 | 0.22 | 0.00 |  |
| S10 | 0.49 | 0.42 | 0.37 | 0.32 | 0.30 | 0.33 | 0.33 | 0.34 | 0.40 | 0.00 |
