# Supplementary material for: ﻿Glacial history of Saxifragawahlenbergii (Saxifragaceae) in the context of refugial areas in the Western Carpathians
Source: PhytoKeys. 2024 Sep 20;246:295–314. doi: 10.3897/phytokeys.246.118796 (PMC11437128; doi:10.3897/phytokeys.246.118796)
Supplement: Supplementary material 5 — Spatial arrangement of varying divergences (FST) among populations within distribution range of Saxifragawahlenbergii [file phytokeys-246-295_article-118796__-s005.pdf]

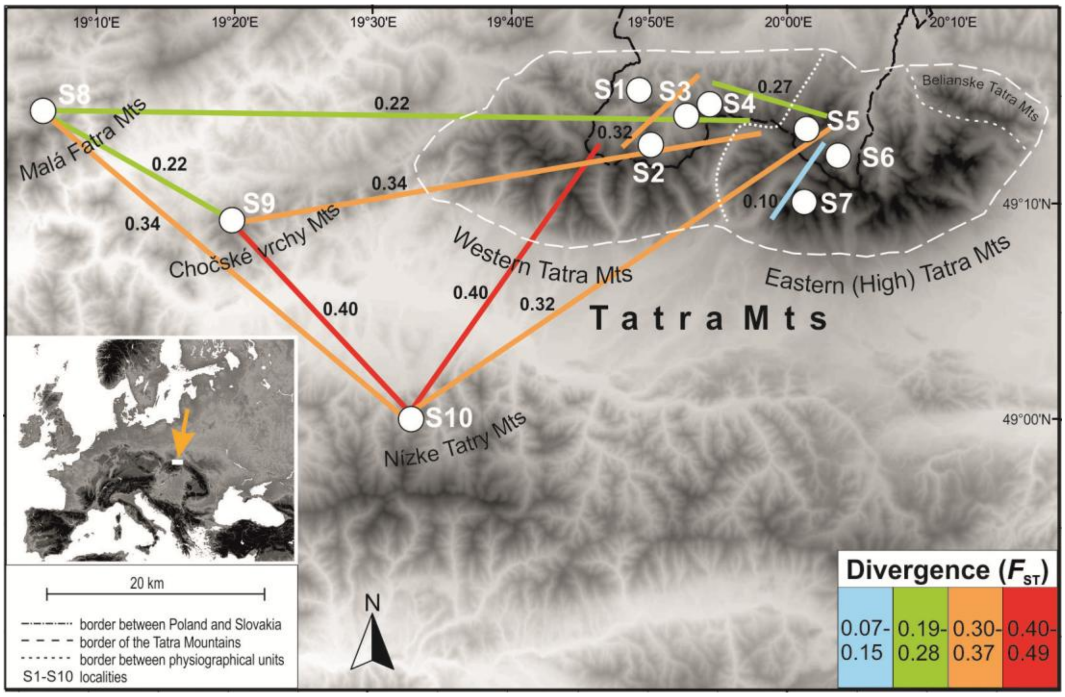

Figure S1. Spatial arrangement of varying divergences (F_{ST}) among populations within distribution range of *Saxifraga wahlenbergii*. Above the line, the average F_{ST} values are given; the line colors correspond to scale in the right corner of the map. For population acronyms see Table 1.
